# (*N*-Ethyl-*N*-phenyl­dithio­carbamato-κ*S*)triphenyl­tin(IV)

**DOI:** 10.1107/S1600536811053591

**Published:** 2011-12-17

**Authors:** Nurul Farahana Kamaludin, Ibrahim Baba, Normah Awang, Mohamed Ibrahim Mohamed Tahir, Edward R. T. Tiekink

**Affiliations:** a Environmental Health Programme, Faculty of Allied Health Sciences, Universiti Kebangsaan Malaysia, Jalan Raja Muda Aziz, 50300 Kuala Lumpur, Malaysia; b School of Chemical Sciences and Food Technology, Faculty of Science and Technology, Universiti Kebangsaan Malaysia, 43600 Bangi, Malaysia; c Department of Chemistry, Universiti Putra Malaysia, 43400 Serdang, Malaysia; d Department of Chemistry, University of Malaya, 50603 Kuala Lumpur, Malaysia

## Abstract

The title compound, [Sn(C_6_H_5_)_3_(C_9_H_10_NS_2_)], has two independent mol­ecules in the asymmetric unit and each features a tetra­hedrally coordinated Sn^IV^ atom as the dithio­carbamate ligand coordinates in a monodentate fashion. As the non-coordinating thione S atom is proximate to the Sn atom [Sn⋯S(thione) = 3.1477 (6) and 2.9970 (5) Å for the independent mol­ecules], distortions from the ideal geometry are evident [the widest angle being 120.48 (5)°]. The most notable feature of the crystal packing is the formation of C—H⋯π inter­actions that lead to the formation of supra­molecular layers parallel to (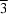
2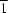).

## Related literature

For a review on the applications and structural chemistry of tin dithio­carbamates, see: Tiekink (2008 ▶). For the recently reported *n*-butyl derivative, see: Kamaludin *et al.* (2011 ▶).
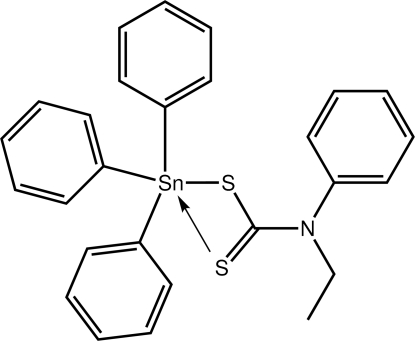


## Experimental

### 

#### Crystal data

[Sn(C_6_H_5_)_3_(C_9_H_10_NS_2_)]
*M* *_r_* = 546.29
Triclinic, 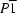

*a* = 9.6973 (2) Å
*b* = 12.2804 (2) Å
*c* = 22.8523 (4) Å
α = 90.588 (2)°
β = 101.573 (2)°
γ = 110.687 (2)°
*V* = 2484.39 (8) Å^3^
*Z* = 4
Mo *K*α radiation
μ = 1.21 mm^−1^
*T* = 150 K
0.30 × 0.24 × 0.19 mm

#### Data collection

Oxford Diffraction Xcaliber Eos Gemini diffractometer
Absorption correction: multi-scan (*CrysAlis PRO*; Oxford Diffraction, 2010 ▶) *T*_min_ = 0.748, *T*_max_ = 0.795
62467 measured reflections
10558 independent reflections
9633 reflections with *I* > 2σ(*I*)
*R*_int_ = 0.038

#### Refinement

*R*[*F* ^2^ > 2σ(*F* ^2^)] = 0.022
*wR*(*F* ^2^) = 0.055
*S* = 1.00
10558 reflections
561 parameters
H-atom parameters constrained
Δρ_max_ = 0.55 e Å^−3^
Δρ_min_ = −0.44 e Å^−3^

### 

Data collection: *CrysAlis PRO* (Oxford Diffraction, 2010 ▶); cell refinement: *CrysAlis PRO*; data reduction: *CrysAlis PRO*; program(s) used to solve structure: *SHELXS97* (Sheldrick, 2008 ▶); program(s) used to refine structure: *SHELXL97* (Sheldrick, 2008 ▶); molecular graphics: *ORTEP-3* (Farrugia, 1997 ▶), *DIAMOND* (Brandenburg, 2006 ▶) and *QMol* (Gans & Shalloway, 2001 ▶); software used to prepare material for publication: *publCIF* (Westrip, 2010 ▶).

## Supplementary Material

Crystal structure: contains datablock(s) global, I. DOI: 10.1107/S1600536811053591/qm2044sup1.cif

Structure factors: contains datablock(s) I. DOI: 10.1107/S1600536811053591/qm2044Isup2.hkl

Additional supplementary materials:  crystallographic information; 3D view; checkCIF report

## Figures and Tables

**Table 1:** Selected bond lengths (Å)

Sn1—C10	2.1339 (19)
Sn1—C16	2.1541 (19)
Sn1—C22	2.1210 (18)
Sn1—S1	2.4539 (5)
Sn2—C37	2.1413 (18)
Sn2—C43	2.1605 (19)
Sn2—C49	2.1379 (19)
Sn2—S3	2.4662 (5)

**Table 2:** Hydrogen-bond geometry (Å, °) *Cg*1, *Cg*2, and *Cg*3 are the centroids of the C16–C21, C37–C42 and C43–C48 benzene rings, respectively.

*D*—H⋯*A*	*D*—H	H⋯*A*	*D*⋯*A*	*D*—H⋯*A*
C9—H9⋯*Cg*1^i^	0.95	2.72	3.630 (3)	160
C25—H25⋯*Cg*2^ii^	0.95	2.90	3.639 (3)	135
C32—H32⋯*Cg*3^iii^	0.95	2.92	3.824 (2)	160

Symmetry codes: (i) 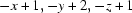; (ii) 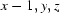; (iii) 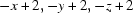.

**Fig. 1.**
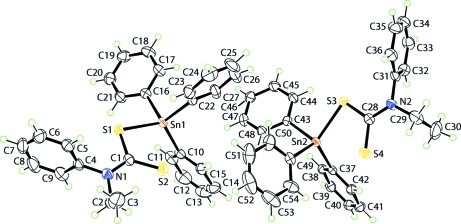
The molecular structure of the two independent molecules comprising (I) showing the atom-labelling scheme and displacement ellipsoids at the 50% probability level.

**Fig. 2.**
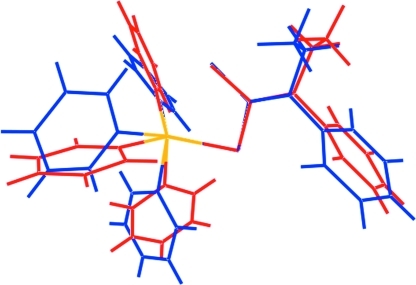
An overlay diagram of the two independent molecules comprising (I) aligned so that the Sn—S—C planes are superimposed. The red image illustrates the molecule containing the Sn1 atom.

**Fig. 3.**
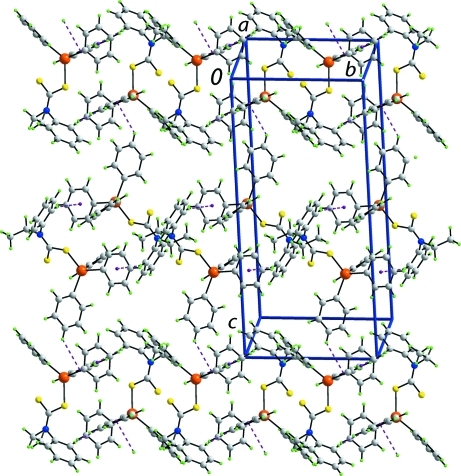
A view of the supramolecular layer parallel to [3 2 1] in (I) mediated by C—H···π interactions (purple dashed lines).

## supplementary crystallographic information

### Comment

Potential applications as anti-cancer agents, anti-microbials and insecticides, and as convenient synthetic precursors for tin sulfide nanoparticles, characterize organotin dithiocarbamates (Tiekink, 2008). This background motivates our interest in this class of compound and led to the investigation of the title compound, (I). Recently, the structure of the *n*-butyl derivative was described (Kamaludin *et al.*, 2011) and herein, we describe the analysis of (I).

There are two independent molecules in the asymmetric unit of (I): the molecular structures are shown in Fig. 1. Each molecule features Sn coordinated by the dithiocarbamate ligand and three *ipso*-C atoms of three benzene rings. The dithiocarbamate ligand coordinates essentially in a monodentate fashion, an assignment supported by the large disparity in the C—S bond distances, Table 1. The coordination geometry is based on a tetrahedron with the range of tetrahedral angles being 94.46 (5) to 120.48 (5)° for the Sn1-containing molecule and 95.28 (5) to 118.66 (5)° for the other. The wider angles are ascribed to the influence of the proximate thione-S atom [Sn1···S2 = 3.1477 (6) Å and Sn2···S4 = 2.9970 (5) Å]. The major differences between the independent molecules is highlighted in the overlay diagram, Fig. 2, showing that the chemically equivalent phenyl rings do not overlap significantly.

The crystal packing of (I) features C—H···π interactions involving the Sn- and *N*-phenyl rings as donors, and Sn-bound phenyl rings as acceptors, Table 2. The result is the formation of supramolecular layers parallel to (3 2 1), Fig. 3.

### Experimental

The title compound was prepared using an *in situ* method. A mixture of ethanol (50 ml) and *N*-ethylaniline (30 m*M*) was added to an ammonia solution (0.25%). The solution was stirred for half an hour at approximately 277 K. Carbon disulfide (30 m*M*) was added drop-wise and stirring was continued for another 6–8 h at 277 K. Triphenytin(IV) chloride (30 m*M*), dissolved in ethanol (20 ml), was added and stirring continued for a further 3 h. The white precipitate formed was filtered, washed with cold ethanol and dried in a vacuum desiccator. Recrystallization was from its ethanol:ethyl acetate (1:1) solution. Yield: 32%. *M*.pt. 381–382 K. Elemental analysis. Found (calculated) for C_27_H_25_NS_2_Sn: C, 59.19 (59.36); H 4.33 (4.61); N 2.52 (2.56); S 11.30 (11.74) %. IR (KBr): ν(C—H) 2986 m; ν(C≐N) 1478 m; ν(N—C) 1125 s; ν(C≐S) 997 s; ν(Sn—S) 357 s cm^-1. 13^C NMR (CDCl_3_): δ (CS_2_) 198.63 p.p.m..

### Refinement

Carbon-bound H-atoms were placed in calculated positions (C—H 0.95 to 0.99 Å) and were included in the refinement in the riding model approximation, with *U*_iso_(H) set to 1.2 to 1.5*U*_equiv_(C).

### Crystal data

**Table:**

[Sn(C_6_H_5_)_3_(C_9_H_10_NS_2_)]	*Z* = 4
*M**_r_* = 546.29	*F*(000) = 1104
Triclinic, *P*1	*D*_x_ = 1.461 Mg m^−^^3^
Hall symbol: -P 1	Mo *K*α radiation, λ = 0.71073 Å
*a* = 9.6973 (2) Å	Cell parameters from 39367 reflections
*b* = 12.2804 (2) Å	θ = 2–29°
*c* = 22.8523 (4) Å	µ = 1.21 mm^−^^1^
α = 90.588 (2)°	*T* = 150 K
β = 101.573 (2)°	Block, colourless
γ = 110.687 (2)°	0.30 × 0.24 × 0.19 mm
*V* = 2484.39 (8) Å^3^	

### Data collection

**Table:**

Oxford Diffraction Xcaliber Eos Gemini diffractometer	10558 independent reflections
Radiation source: fine-focus sealed tube	9633 reflections with *I* > 2σ(*I*)
graphite	*R*_int_ = 0.038
Detector resolution: 16.1952 pixels mm^-1^	θ_max_ = 26.8°, θ_min_ = 2.3°
ω scans	*h* = −12→12
Absorption correction: multi-scan (*CrysAlis PRO*; Oxford Diffraction, 2010)	*k* = −15→15
*T*_min_ = 0.748, *T*_max_ = 0.795	*l* = −28→28
62467 measured reflections	

### Refinement

**Table:**

Refinement on *F*^2^	Primary atom site location: structure-invariant direct methods
Least-squares matrix: full	Secondary atom site location: difference Fourier map
*R*[*F*^2^ > 2σ(*F*^2^)] = 0.022	Hydrogen site location: inferred from neighbouring sites
*wR*(*F*^2^) = 0.055	H-atom parameters constrained
*S* = 1.00	*w* = 1/[σ^2^(*F*_o_^2^) + (0.0259*P*)^2^ + 1.489*P*] where *P* = (*F*_o_^2^ + 2*F*_c_^2^)/3
10558 reflections	(Δ/σ)_max_ = 0.004
561 parameters	Δρ_max_ = 0.55 e Å^−^^3^
0 restraints	Δρ_min_ = −0.44 e Å^−^^3^

### Special details

**Table:**

Geometry. All s.u.'s (except the s.u. in the dihedral angle between two l.s. planes) are estimated using the full covariance matrix. The cell s.u.'s are taken into account individually in the estimation of s.u.'s in distances, angles and torsion angles; correlations between s.u.'s in cell parameters are only used when they are defined by crystal symmetry. An approximate (isotropic) treatment of cell s.u.'s is used for estimating s.u.'s involving l.s. planes.
Refinement. Refinement of *F*^2^ against ALL reflections. The weighted *R*-factor *wR* and goodness of fit *S* are based on *F*^2^, conventional *R*-factors *R* are based on *F*, with *F* set to zero for negative *F*^2^. The threshold expression of *F*^2^ > 2σ(*F*^2^) is used only for calculating *R*-factors(gt) *etc*. and is not relevant to the choice of reflections for refinement. *R*-factors based on *F*^2^ are statistically about twice as large as those based on *F*, and *R*- factors based on ALL data will be even larger.

### Fractional atomic coordinates and isotropic or equivalent isotropic displacement parameters (Å^2^)

**Table:**

	*x*	*y*	*z*	*U*_iso_*/*U*_eq_	
Sn1	0.425600 (14)	0.868539 (11)	0.613068 (5)	0.02407 (4)	
S1	0.25460 (6)	0.77901 (4)	0.51651 (2)	0.02845 (10)	
S2	0.39527 (6)	0.61380 (5)	0.57263 (2)	0.03395 (11)	
N1	0.2447 (2)	0.58873 (15)	0.45887 (7)	0.0337 (4)	
C1	0.2955 (2)	0.65087 (17)	0.51249 (8)	0.0284 (4)	
C2	0.2836 (3)	0.4851 (2)	0.44586 (11)	0.0478 (6)	
H2A	0.3831	0.4944	0.4715	0.057*	
H2B	0.2923	0.4815	0.4035	0.057*	
C3	0.1682 (3)	0.3731 (2)	0.45672 (13)	0.0614 (8)	
H3A	0.1678	0.3723	0.4996	0.092*	
H3B	0.1931	0.3071	0.4439	0.092*	
H3C	0.0682	0.3663	0.4338	0.092*	
C4	0.1517 (2)	0.62092 (17)	0.40950 (8)	0.0314 (4)	
C5	−0.0031 (3)	0.5806 (2)	0.40371 (10)	0.0386 (5)	
H5	−0.0482	0.5333	0.4324	0.046*	
C6	−0.0915 (3)	0.6096 (2)	0.35598 (11)	0.0510 (6)	
H6	−0.1980	0.5823	0.3517	0.061*	
C7	−0.0259 (4)	0.6780 (3)	0.31455 (11)	0.0610 (8)	
H7	−0.0871	0.6985	0.2819	0.073*	
C8	0.1281 (4)	0.7169 (2)	0.32016 (11)	0.0602 (8)	
H8	0.1725	0.7636	0.2911	0.072*	
C9	0.2197 (3)	0.6885 (2)	0.36788 (10)	0.0440 (5)	
H9	0.3260	0.7149	0.3717	0.053*	
C10	0.6605 (2)	0.89709 (18)	0.62716 (8)	0.0293 (4)	
C11	0.7603 (2)	0.9959 (2)	0.60893 (10)	0.0408 (5)	
H11	0.7239	1.0507	0.5885	0.049*	
C12	0.9148 (3)	1.0149 (3)	0.62057 (13)	0.0574 (7)	
H12	0.9828	1.0815	0.6072	0.069*	
C13	0.9681 (3)	0.9371 (3)	0.65134 (14)	0.0624 (8)	
H13	1.0730	0.9503	0.6591	0.075*	
C14	0.8709 (3)	0.8402 (2)	0.67092 (12)	0.0517 (7)	
H14	0.9086	0.7877	0.6929	0.062*	
C15	0.7171 (2)	0.8197 (2)	0.65846 (9)	0.0359 (5)	
H15	0.6499	0.7521	0.6714	0.043*	
C16	0.4002 (2)	1.03524 (16)	0.60391 (8)	0.0271 (4)	
C17	0.3810 (2)	1.09529 (17)	0.65201 (9)	0.0332 (4)	
H17	0.3829	1.0632	0.6897	0.040*	
C18	0.3592 (3)	1.20067 (19)	0.64607 (10)	0.0408 (5)	
H18	0.3465	1.2399	0.6795	0.049*	
C19	0.3559 (3)	1.24854 (19)	0.59177 (11)	0.0421 (5)	
H19	0.3412	1.3208	0.5877	0.051*	
C20	0.3740 (3)	1.1909 (2)	0.54327 (10)	0.0415 (5)	
H20	0.3716	1.2235	0.5057	0.050*	
C21	0.3956 (2)	1.08579 (19)	0.54950 (9)	0.0350 (5)	
H21	0.4077	1.0469	0.5158	0.042*	
C22	0.3337 (2)	0.80327 (17)	0.68794 (8)	0.0268 (4)	
C23	0.1960 (2)	0.7136 (2)	0.68340 (10)	0.0386 (5)	
H23	0.1416	0.6716	0.6457	0.046*	
C24	0.1371 (3)	0.6849 (2)	0.73484 (14)	0.0581 (7)	
H24	0.0423	0.6237	0.7321	0.070*	
C25	0.2182 (4)	0.7465 (3)	0.78978 (12)	0.0635 (8)	
H25	0.1774	0.7282	0.8245	0.076*	
C26	0.3555 (4)	0.8328 (2)	0.79434 (11)	0.0593 (8)	
H26	0.4117	0.8730	0.8322	0.071*	
C27	0.4122 (3)	0.8612 (2)	0.74395 (9)	0.0410 (5)	
H27	0.5076	0.9221	0.7474	0.049*	
Sn2	0.849644 (14)	0.767651 (10)	0.914470 (5)	0.02217 (4)	
S3	0.73045 (6)	0.77465 (4)	0.99945 (2)	0.02867 (10)	
S4	0.82367 (6)	0.56918 (4)	0.99206 (2)	0.03252 (11)	
N2	0.71753 (19)	0.62538 (13)	1.08192 (7)	0.0289 (3)	
C28	0.7555 (2)	0.65068 (16)	1.02941 (8)	0.0257 (4)	
C29	0.7378 (3)	0.52486 (19)	1.11280 (11)	0.0483 (6)	
H29A	0.7194	0.4601	1.0826	0.058*	
H29B	0.6630	0.4970	1.1382	0.058*	
C30	0.8959 (4)	0.5582 (3)	1.15146 (13)	0.0726 (9)	
H30A	0.9696	0.5783	1.1258	0.109*	
H30B	0.9035	0.4921	1.1738	0.109*	
H30C	0.9168	0.6256	1.1797	0.109*	
C31	0.6641 (2)	0.69863 (16)	1.11445 (8)	0.0273 (4)	
C32	0.7662 (2)	0.79839 (17)	1.14875 (9)	0.0335 (4)	
H32	0.8711	0.8217	1.1497	0.040*	
C33	0.7126 (3)	0.86425 (19)	1.18188 (10)	0.0409 (5)	
H33	0.7811	0.9330	1.2059	0.049*	
C34	0.5593 (3)	0.8294 (2)	1.17981 (10)	0.0420 (5)	
H34	0.5227	0.8747	1.2022	0.050*	
C35	0.4607 (3)	0.7303 (2)	1.14582 (11)	0.0440 (5)	
H35	0.3557	0.7072	1.1447	0.053*	
C36	0.5116 (2)	0.66293 (19)	1.11289 (10)	0.0360 (5)	
H36	0.4427	0.5934	1.0897	0.043*	
C37	1.0884 (2)	0.80657 (15)	0.93488 (8)	0.0225 (4)	
C38	1.1777 (2)	0.91710 (16)	0.92315 (8)	0.0251 (4)	
H38	1.1314	0.9708	0.9084	0.030*	
C39	1.3336 (2)	0.95029 (17)	0.93266 (8)	0.0297 (4)	
H39	1.3931	1.0261	0.9244	0.036*	
C40	1.4021 (2)	0.87260 (18)	0.95422 (9)	0.0313 (4)	
H40	1.5086	0.8947	0.9605	0.038*	
C41	1.3150 (2)	0.76280 (18)	0.96660 (9)	0.0320 (4)	
H41	1.3621	0.7099	0.9818	0.038*	
C42	1.1587 (2)	0.72925 (17)	0.95689 (8)	0.0284 (4)	
H42	1.0997	0.6534	0.9653	0.034*	
C43	0.8300 (2)	0.92594 (16)	0.88000 (8)	0.0249 (4)	
C44	0.8243 (2)	1.01716 (18)	0.91533 (9)	0.0329 (4)	
H44	0.8266	1.0101	0.9568	0.040*	
C45	0.8153 (3)	1.1176 (2)	0.89075 (10)	0.0413 (5)	
H45	0.8109	1.1786	0.9154	0.050*	
C46	0.8125 (2)	1.12962 (18)	0.83056 (9)	0.0350 (5)	
H46	0.8052	1.1983	0.8137	0.042*	
C47	0.8206 (2)	1.04148 (18)	0.79481 (9)	0.0330 (4)	
H47	0.8205	1.0499	0.7535	0.040*	
C48	0.8287 (2)	0.94077 (17)	0.81961 (8)	0.0300 (4)	
H48	0.8335	0.8803	0.7947	0.036*	
C49	0.7240 (2)	0.64207 (16)	0.83962 (8)	0.0285 (4)	
C50	0.5873 (3)	0.6435 (2)	0.80722 (10)	0.0440 (5)	
H50	0.5436	0.6937	0.8215	0.053*	
C51	0.5138 (3)	0.5727 (2)	0.75430 (12)	0.0630 (8)	
H51	0.4197	0.5741	0.7329	0.076*	
C52	0.5760 (4)	0.5013 (2)	0.73294 (12)	0.0695 (10)	
H52	0.5261	0.4539	0.6963	0.083*	
C53	0.7108 (4)	0.4976 (2)	0.76434 (13)	0.0670 (9)	
H53	0.7537	0.4474	0.7494	0.080*	
C54	0.7849 (3)	0.5676 (2)	0.81818 (11)	0.0456 (6)	
H54	0.8772	0.5639	0.8401	0.055*	

### Atomic displacement parameters (Å^2^)

**Table:**

	*U*^11^	*U*^22^	*U*^33^	*U*^12^	*U*^13^	*U*^23^
Sn1	0.02483 (7)	0.02598 (7)	0.01999 (7)	0.00680 (5)	0.00633 (5)	0.00045 (5)
S1	0.0337 (3)	0.0290 (2)	0.0217 (2)	0.0118 (2)	0.00338 (19)	−0.00109 (18)
S2	0.0372 (3)	0.0334 (3)	0.0284 (2)	0.0131 (2)	0.0004 (2)	0.0015 (2)
N1	0.0406 (10)	0.0317 (9)	0.0273 (8)	0.0143 (8)	0.0021 (7)	−0.0043 (7)
C1	0.0275 (10)	0.0282 (10)	0.0256 (9)	0.0054 (8)	0.0061 (8)	0.0005 (7)
C2	0.0574 (15)	0.0529 (15)	0.0401 (13)	0.0336 (13)	0.0016 (11)	−0.0077 (11)
C3	0.0743 (19)	0.0410 (14)	0.0637 (18)	0.0265 (14)	−0.0058 (15)	−0.0103 (12)
C4	0.0423 (12)	0.0292 (10)	0.0209 (9)	0.0136 (9)	0.0017 (8)	−0.0057 (7)
C5	0.0437 (13)	0.0378 (12)	0.0331 (11)	0.0155 (10)	0.0043 (9)	−0.0046 (9)
C6	0.0518 (15)	0.0561 (15)	0.0439 (14)	0.0285 (13)	−0.0079 (11)	−0.0133 (12)
C7	0.094 (2)	0.0664 (18)	0.0312 (13)	0.0537 (18)	−0.0121 (14)	−0.0081 (12)
C8	0.105 (3)	0.0555 (16)	0.0303 (12)	0.0382 (17)	0.0208 (14)	0.0141 (11)
C9	0.0528 (14)	0.0423 (13)	0.0371 (12)	0.0143 (11)	0.0156 (11)	0.0035 (10)
C10	0.0262 (10)	0.0353 (11)	0.0235 (9)	0.0072 (8)	0.0067 (7)	−0.0044 (8)
C11	0.0342 (11)	0.0423 (12)	0.0364 (12)	0.0015 (10)	0.0102 (9)	−0.0028 (9)
C12	0.0339 (13)	0.0641 (17)	0.0583 (16)	−0.0058 (12)	0.0190 (12)	−0.0106 (13)
C13	0.0251 (12)	0.085 (2)	0.0691 (18)	0.0136 (13)	0.0054 (12)	−0.0264 (16)
C14	0.0388 (13)	0.0656 (17)	0.0501 (14)	0.0266 (13)	−0.0048 (11)	−0.0192 (12)
C15	0.0327 (11)	0.0408 (12)	0.0313 (11)	0.0131 (9)	0.0017 (8)	−0.0062 (9)
C16	0.0255 (9)	0.0253 (9)	0.0284 (10)	0.0057 (8)	0.0077 (8)	0.0004 (7)
C17	0.0392 (11)	0.0293 (10)	0.0294 (10)	0.0074 (9)	0.0133 (9)	0.0011 (8)
C18	0.0484 (13)	0.0341 (11)	0.0426 (12)	0.0136 (10)	0.0187 (10)	−0.0035 (9)
C19	0.0488 (13)	0.0317 (11)	0.0518 (14)	0.0192 (10)	0.0158 (11)	0.0055 (10)
C20	0.0521 (14)	0.0416 (12)	0.0377 (12)	0.0227 (11)	0.0139 (10)	0.0119 (10)
C21	0.0427 (12)	0.0358 (11)	0.0295 (10)	0.0157 (10)	0.0119 (9)	0.0030 (8)
C22	0.0322 (10)	0.0313 (10)	0.0222 (9)	0.0163 (8)	0.0085 (8)	0.0059 (7)
C23	0.0366 (12)	0.0419 (12)	0.0376 (12)	0.0122 (10)	0.0116 (9)	0.0128 (9)
C24	0.0554 (16)	0.0576 (16)	0.075 (2)	0.0238 (14)	0.0380 (15)	0.0376 (15)
C25	0.106 (3)	0.0722 (19)	0.0443 (15)	0.0525 (19)	0.0494 (17)	0.0245 (14)
C26	0.105 (2)	0.0549 (16)	0.0273 (12)	0.0363 (17)	0.0212 (14)	0.0058 (11)
C27	0.0556 (14)	0.0401 (12)	0.0254 (10)	0.0173 (11)	0.0050 (10)	0.0002 (9)
Sn2	0.02496 (7)	0.02192 (7)	0.01935 (6)	0.00797 (5)	0.00520 (5)	0.00102 (5)
S3	0.0357 (3)	0.0334 (3)	0.0260 (2)	0.0199 (2)	0.0129 (2)	0.00964 (19)
S4	0.0459 (3)	0.0260 (2)	0.0315 (3)	0.0154 (2)	0.0169 (2)	0.00464 (19)
N2	0.0399 (9)	0.0213 (8)	0.0287 (8)	0.0100 (7)	0.0164 (7)	0.0061 (6)
C28	0.0257 (9)	0.0238 (9)	0.0252 (9)	0.0054 (8)	0.0066 (7)	0.0018 (7)
C29	0.0849 (19)	0.0261 (11)	0.0492 (14)	0.0252 (12)	0.0386 (14)	0.0172 (10)
C30	0.122 (3)	0.0634 (19)	0.0487 (16)	0.059 (2)	0.0063 (17)	0.0182 (14)
C31	0.0375 (11)	0.0254 (9)	0.0216 (9)	0.0116 (8)	0.0119 (8)	0.0063 (7)
C32	0.0340 (11)	0.0281 (10)	0.0365 (11)	0.0086 (9)	0.0081 (9)	0.0031 (8)
C33	0.0569 (15)	0.0311 (11)	0.0318 (11)	0.0141 (10)	0.0072 (10)	−0.0033 (9)
C34	0.0608 (15)	0.0445 (13)	0.0341 (11)	0.0281 (12)	0.0233 (11)	0.0073 (10)
C35	0.0404 (13)	0.0488 (14)	0.0498 (14)	0.0173 (11)	0.0231 (11)	0.0099 (11)
C36	0.0363 (11)	0.0335 (11)	0.0346 (11)	0.0063 (9)	0.0113 (9)	0.0027 (9)
C37	0.0259 (9)	0.0247 (9)	0.0173 (8)	0.0100 (7)	0.0042 (7)	−0.0011 (7)
C38	0.0295 (10)	0.0253 (9)	0.0210 (9)	0.0117 (8)	0.0031 (7)	0.0005 (7)
C39	0.0287 (10)	0.0296 (10)	0.0259 (9)	0.0052 (8)	0.0050 (8)	0.0004 (8)
C40	0.0258 (10)	0.0387 (11)	0.0267 (10)	0.0117 (9)	0.0002 (8)	−0.0069 (8)
C41	0.0349 (11)	0.0341 (11)	0.0290 (10)	0.0190 (9)	−0.0002 (8)	−0.0031 (8)
C42	0.0346 (10)	0.0243 (9)	0.0272 (9)	0.0123 (8)	0.0054 (8)	0.0007 (7)
C43	0.0243 (9)	0.0270 (9)	0.0234 (9)	0.0101 (8)	0.0038 (7)	0.0019 (7)
C44	0.0471 (12)	0.0356 (11)	0.0222 (9)	0.0210 (10)	0.0098 (9)	0.0026 (8)
C45	0.0644 (15)	0.0368 (12)	0.0362 (12)	0.0303 (11)	0.0188 (11)	0.0054 (9)
C46	0.0422 (12)	0.0311 (11)	0.0366 (11)	0.0179 (9)	0.0105 (9)	0.0110 (9)
C47	0.0391 (11)	0.0333 (11)	0.0224 (9)	0.0100 (9)	0.0034 (8)	0.0052 (8)
C48	0.0391 (11)	0.0259 (10)	0.0228 (9)	0.0090 (8)	0.0071 (8)	−0.0003 (7)
C49	0.0334 (10)	0.0222 (9)	0.0230 (9)	0.0011 (8)	0.0072 (8)	0.0007 (7)
C50	0.0428 (13)	0.0346 (12)	0.0420 (13)	0.0060 (10)	−0.0040 (10)	0.0021 (10)
C51	0.0662 (18)	0.0442 (15)	0.0438 (14)	−0.0060 (13)	−0.0172 (13)	0.0028 (12)
C52	0.092 (2)	0.0412 (15)	0.0336 (13)	−0.0187 (15)	0.0006 (14)	−0.0061 (11)
C53	0.095 (2)	0.0418 (15)	0.0559 (17)	0.0058 (15)	0.0331 (17)	−0.0173 (13)
C54	0.0497 (14)	0.0393 (13)	0.0450 (13)	0.0111 (11)	0.0139 (11)	−0.0103 (10)

### Geometric parameters (Å, °)

**Table:**

Sn1—C10	2.1339 (19)	Sn2—C37	2.1413 (18)
Sn1—C16	2.1541 (19)	Sn2—C43	2.1605 (19)
Sn1—C22	2.1210 (18)	Sn2—C49	2.1379 (19)
Sn1—S1	2.4539 (5)	Sn2—S3	2.4662 (5)
S1—C1	1.759 (2)	S3—C28	1.7496 (19)
S2—C1	1.680 (2)	S4—C28	1.6862 (19)
N1—C1	1.342 (2)	N2—C28	1.333 (2)
N1—C4	1.448 (3)	N2—C31	1.449 (2)
N1—C2	1.492 (3)	N2—C29	1.481 (3)
C2—C3	1.496 (4)	C29—C30	1.518 (4)
C2—H2A	0.9900	C29—H29A	0.9900
C2—H2B	0.9900	C29—H29B	0.9900
C3—H3A	0.9800	C30—H30A	0.9800
C3—H3B	0.9800	C30—H30B	0.9800
C3—H3C	0.9800	C30—H30C	0.9800
C4—C9	1.382 (3)	C31—C32	1.381 (3)
C4—C5	1.382 (3)	C31—C36	1.379 (3)
C5—C6	1.379 (3)	C32—C33	1.393 (3)
C5—H5	0.9500	C32—H32	0.9500
C6—C7	1.374 (4)	C33—C34	1.384 (3)
C6—H6	0.9500	C33—H33	0.9500
C7—C8	1.376 (4)	C34—C35	1.363 (3)
C7—H7	0.9500	C34—H34	0.9500
C8—C9	1.391 (4)	C35—C36	1.386 (3)
C8—H8	0.9500	C35—H35	0.9500
C9—H9	0.9500	C36—H36	0.9500
C10—C15	1.393 (3)	C37—C38	1.391 (3)
C10—C11	1.388 (3)	C37—C42	1.396 (3)
C11—C12	1.400 (3)	C38—C39	1.389 (3)
C11—H11	0.9500	C38—H38	0.9500
C12—C13	1.375 (4)	C39—C40	1.386 (3)
C12—H12	0.9500	C39—H39	0.9500
C13—C14	1.375 (4)	C40—C41	1.382 (3)
C13—H13	0.9500	C40—H40	0.9500
C14—C15	1.389 (3)	C41—C42	1.392 (3)
C14—H14	0.9500	C41—H41	0.9500
C15—H15	0.9500	C42—H42	0.9500
C16—C17	1.396 (3)	C43—C48	1.392 (3)
C16—C21	1.395 (3)	C43—C44	1.398 (3)
C17—C18	1.387 (3)	C44—C45	1.384 (3)
C17—H17	0.9500	C44—H44	0.9500
C18—C19	1.378 (3)	C45—C46	1.381 (3)
C18—H18	0.9500	C45—H45	0.9500
C19—C20	1.382 (3)	C46—C47	1.383 (3)
C19—H19	0.9500	C46—H46	0.9500
C20—C21	1.384 (3)	C47—C48	1.386 (3)
C20—H20	0.9500	C47—H47	0.9500
C21—H21	0.9500	C48—H48	0.9500
C22—C27	1.390 (3)	C49—C50	1.389 (3)
C22—C23	1.382 (3)	C49—C54	1.384 (3)
C23—C24	1.403 (3)	C50—C51	1.386 (3)
C23—H23	0.9500	C50—H50	0.9500
C24—C25	1.390 (4)	C51—C52	1.362 (5)
C24—H24	0.9500	C51—H51	0.9500
C25—C26	1.361 (4)	C52—C53	1.376 (5)
C25—H25	0.9500	C52—H52	0.9500
C26—C27	1.370 (3)	C53—C54	1.398 (4)
C26—H26	0.9500	C53—H53	0.9500
C27—H27	0.9500	C54—H54	0.9500
			
C22—Sn1—C10	112.49 (7)	C49—Sn2—C37	115.41 (7)
C22—Sn1—C16	105.17 (7)	C49—Sn2—C43	101.51 (7)
C10—Sn1—C16	108.02 (8)	C37—Sn2—C43	103.22 (7)
C22—Sn1—S1	113.28 (6)	C49—Sn2—S3	118.66 (5)
C10—Sn1—S1	120.48 (5)	C37—Sn2—S3	117.12 (5)
C16—Sn1—S1	94.46 (5)	C43—Sn2—S3	95.28 (5)
C1—S1—Sn1	98.20 (7)	C28—S3—Sn2	95.09 (6)
C1—N1—C4	121.45 (17)	C28—N2—C31	122.39 (15)
C1—N1—C2	122.48 (18)	C28—N2—C29	121.82 (16)
C4—N1—C2	116.05 (16)	C31—N2—C29	115.65 (15)
N1—C1—S2	123.77 (16)	N2—C28—S4	123.09 (14)
N1—C1—S1	115.59 (15)	N2—C28—S3	116.67 (14)
S2—C1—S1	120.63 (11)	S4—C28—S3	120.24 (11)
N1—C2—C3	112.3 (2)	N2—C29—C30	111.3 (2)
N1—C2—H2A	109.1	N2—C29—H29A	109.4
C3—C2—H2A	109.1	C30—C29—H29A	109.4
N1—C2—H2B	109.1	N2—C29—H29B	109.4
C3—C2—H2B	109.1	C30—C29—H29B	109.4
H2A—C2—H2B	107.9	H29A—C29—H29B	108.0
C2—C3—H3A	109.5	C29—C30—H30A	109.5
C2—C3—H3B	109.5	C29—C30—H30B	109.5
H3A—C3—H3B	109.5	H30A—C30—H30B	109.5
C2—C3—H3C	109.5	C29—C30—H30C	109.5
H3A—C3—H3C	109.5	H30A—C30—H30C	109.5
H3B—C3—H3C	109.5	H30B—C30—H30C	109.5
C9—C4—C5	121.3 (2)	C32—C31—C36	121.41 (19)
C9—C4—N1	119.0 (2)	C32—C31—N2	119.92 (18)
C5—C4—N1	119.70 (19)	C36—C31—N2	118.56 (18)
C6—C5—C4	119.5 (2)	C31—C32—C33	118.8 (2)
C6—C5—H5	120.2	C31—C32—H32	120.6
C4—C5—H5	120.2	C33—C32—H32	120.6
C7—C6—C5	120.1 (3)	C34—C33—C32	119.9 (2)
C7—C6—H6	120.0	C34—C33—H33	120.0
C5—C6—H6	120.0	C32—C33—H33	120.0
C8—C7—C6	120.2 (2)	C35—C34—C33	120.3 (2)
C8—C7—H7	119.9	C35—C34—H34	119.9
C6—C7—H7	119.9	C33—C34—H34	119.9
C7—C8—C9	120.8 (3)	C34—C35—C36	120.8 (2)
C7—C8—H8	119.6	C34—C35—H35	119.6
C9—C8—H8	119.6	C36—C35—H35	119.6
C4—C9—C8	118.2 (2)	C31—C36—C35	118.8 (2)
C4—C9—H9	120.9	C31—C36—H36	120.6
C8—C9—H9	120.9	C35—C36—H36	120.6
C15—C10—C11	118.9 (2)	C38—C37—C42	118.60 (17)
C15—C10—Sn1	119.93 (15)	C38—C37—Sn2	116.12 (13)
C11—C10—Sn1	121.02 (16)	C42—C37—Sn2	125.26 (14)
C10—C11—C12	120.0 (2)	C39—C38—C37	121.05 (17)
C10—C11—H11	120.0	C39—C38—H38	119.5
C12—C11—H11	120.0	C37—C38—H38	119.5
C13—C12—C11	120.0 (3)	C38—C39—C40	119.83 (19)
C13—C12—H12	120.0	C38—C39—H39	120.1
C11—C12—H12	120.0	C40—C39—H39	120.1
C12—C13—C14	120.6 (2)	C41—C40—C39	119.82 (19)
C12—C13—H13	119.7	C41—C40—H40	120.1
C14—C13—H13	119.7	C39—C40—H40	120.1
C13—C14—C15	119.7 (3)	C40—C41—C42	120.42 (18)
C13—C14—H14	120.2	C40—C41—H41	119.8
C15—C14—H14	120.2	C42—C41—H41	119.8
C10—C15—C14	120.8 (2)	C41—C42—C37	120.28 (18)
C10—C15—H15	119.6	C41—C42—H42	119.9
C14—C15—H15	119.6	C37—C42—H42	119.9
C17—C16—C21	116.95 (18)	C48—C43—C44	117.55 (17)
C17—C16—Sn1	121.01 (14)	C48—C43—Sn2	118.58 (13)
C21—C16—Sn1	121.99 (14)	C44—C43—Sn2	123.82 (14)
C18—C17—C16	121.53 (19)	C45—C44—C43	121.00 (18)
C18—C17—H17	119.2	C45—C44—H44	119.5
C16—C17—H17	119.2	C43—C44—H44	119.5
C19—C18—C17	120.1 (2)	C44—C45—C46	120.29 (19)
C19—C18—H18	120.0	C44—C45—H45	119.9
C17—C18—H18	120.0	C46—C45—H45	119.9
C20—C19—C18	119.7 (2)	C47—C46—C45	119.88 (19)
C20—C19—H19	120.1	C47—C46—H46	120.1
C18—C19—H19	120.1	C45—C46—H46	120.1
C19—C20—C21	119.9 (2)	C46—C47—C48	119.56 (18)
C19—C20—H20	120.1	C46—C47—H47	120.2
C21—C20—H20	120.1	C48—C47—H47	120.2
C20—C21—C16	121.82 (19)	C43—C48—C47	121.71 (18)
C20—C21—H21	119.1	C43—C48—H48	119.1
C16—C21—H21	119.1	C47—C48—H48	119.1
C27—C22—C23	118.84 (19)	C50—C49—C54	118.5 (2)
C27—C22—Sn1	117.20 (16)	C50—C49—Sn2	120.19 (16)
C23—C22—Sn1	123.75 (15)	C54—C49—Sn2	120.84 (16)
C22—C23—C24	119.6 (2)	C51—C50—C49	120.9 (3)
C22—C23—H23	120.2	C51—C50—H50	119.5
C24—C23—H23	120.2	C49—C50—H50	119.5
C25—C24—C23	119.7 (3)	C52—C51—C50	120.1 (3)
C25—C24—H24	120.2	C52—C51—H51	119.9
C23—C24—H24	120.2	C50—C51—H51	119.9
C26—C25—C24	120.6 (2)	C51—C52—C53	120.1 (2)
C26—C25—H25	119.7	C51—C52—H52	119.9
C24—C25—H25	119.7	C53—C52—H52	119.9
C25—C26—C27	119.6 (3)	C52—C53—C54	120.2 (3)
C25—C26—H26	120.2	C52—C53—H53	119.9
C27—C26—H26	120.2	C54—C53—H53	119.9
C26—C27—C22	121.7 (2)	C53—C54—C49	120.1 (3)
C26—C27—H27	119.1	C53—C54—H54	120.0
C22—C27—H27	119.1	C49—C54—H54	120.0
			
C22—Sn1—S1—C1	−82.34 (8)	C49—Sn2—S3—C28	76.70 (9)
C10—Sn1—S1—C1	55.09 (9)	C37—Sn2—S3—C28	−69.32 (8)
C16—Sn1—S1—C1	169.04 (8)	C43—Sn2—S3—C28	−177.10 (8)
C4—N1—C1—S2	176.31 (16)	C31—N2—C28—S4	177.77 (15)
C2—N1—C1—S2	−5.4 (3)	C29—N2—C28—S4	2.3 (3)
C4—N1—C1—S1	−5.0 (3)	C31—N2—C28—S3	−2.6 (3)
C2—N1—C1—S1	173.26 (17)	C29—N2—C28—S3	−178.10 (17)
Sn1—S1—C1—N1	−166.86 (14)	Sn2—S3—C28—N2	174.02 (14)
Sn1—S1—C1—S2	11.88 (12)	Sn2—S3—C28—S4	−6.32 (12)
C1—N1—C2—C3	92.9 (3)	C28—N2—C29—C30	86.3 (3)
C4—N1—C2—C3	−88.7 (2)	C31—N2—C29—C30	−89.5 (2)
C1—N1—C4—C9	95.5 (2)	C28—N2—C31—C32	−80.5 (2)
C2—N1—C4—C9	−82.9 (3)	C29—N2—C31—C32	95.2 (2)
C1—N1—C4—C5	−86.6 (2)	C28—N2—C31—C36	103.0 (2)
C2—N1—C4—C5	95.0 (2)	C29—N2—C31—C36	−81.2 (2)
C9—C4—C5—C6	−0.9 (3)	C36—C31—C32—C33	−0.6 (3)
N1—C4—C5—C6	−178.75 (19)	N2—C31—C32—C33	−176.94 (18)
C4—C5—C6—C7	0.0 (3)	C31—C32—C33—C34	−0.2 (3)
C5—C6—C7—C8	0.7 (4)	C32—C33—C34—C35	0.5 (3)
C6—C7—C8—C9	−0.5 (4)	C33—C34—C35—C36	0.0 (4)
C5—C4—C9—C8	1.0 (3)	C32—C31—C36—C35	1.1 (3)
N1—C4—C9—C8	178.9 (2)	N2—C31—C36—C35	177.50 (19)
C7—C8—C9—C4	−0.3 (4)	C34—C35—C36—C31	−0.8 (3)
C22—Sn1—C10—C15	37.46 (17)	C49—Sn2—C37—C38	109.91 (13)
C16—Sn1—C10—C15	153.09 (15)	C43—Sn2—C37—C38	0.12 (14)
S1—Sn1—C10—C15	−100.26 (15)	S3—Sn2—C37—C38	−102.97 (13)
C22—Sn1—C10—C11	−138.42 (16)	C49—Sn2—C37—C42	−68.03 (17)
C16—Sn1—C10—C11	−22.79 (18)	C43—Sn2—C37—C42	−177.83 (15)
S1—Sn1—C10—C11	83.85 (16)	S3—Sn2—C37—C42	79.09 (15)
C15—C10—C11—C12	1.8 (3)	C42—C37—C38—C39	0.4 (3)
Sn1—C10—C11—C12	177.71 (17)	Sn2—C37—C38—C39	−177.68 (14)
C10—C11—C12—C13	−1.6 (4)	C37—C38—C39—C40	−0.1 (3)
C11—C12—C13—C14	0.0 (4)	C38—C39—C40—C41	−0.5 (3)
C12—C13—C14—C15	1.3 (4)	C39—C40—C41—C42	0.7 (3)
C11—C10—C15—C14	−0.5 (3)	C40—C41—C42—C37	−0.4 (3)
Sn1—C10—C15—C14	−176.47 (16)	C38—C37—C42—C41	−0.2 (3)
C13—C14—C15—C10	−1.0 (3)	Sn2—C37—C42—C41	177.73 (14)
C22—Sn1—C16—C17	20.76 (18)	C49—Sn2—C43—C48	−35.27 (16)
C10—Sn1—C16—C17	−99.57 (17)	C37—Sn2—C43—C48	84.57 (16)
S1—Sn1—C16—C17	136.35 (16)	S3—Sn2—C43—C48	−155.95 (15)
C22—Sn1—C16—C21	−156.46 (16)	C49—Sn2—C43—C44	147.26 (17)
C10—Sn1—C16—C21	83.20 (17)	C37—Sn2—C43—C44	−92.89 (17)
S1—Sn1—C16—C21	−40.88 (17)	S3—Sn2—C43—C44	26.58 (17)
C21—C16—C17—C18	−0.3 (3)	C48—C43—C44—C45	1.0 (3)
Sn1—C16—C17—C18	−177.70 (17)	Sn2—C43—C44—C45	178.45 (17)
C16—C17—C18—C19	0.1 (3)	C43—C44—C45—C46	−0.3 (4)
C17—C18—C19—C20	0.2 (4)	C44—C45—C46—C47	−0.7 (4)
C18—C19—C20—C21	−0.1 (4)	C45—C46—C47—C48	1.1 (3)
C19—C20—C21—C16	−0.1 (4)	C44—C43—C48—C47	−0.6 (3)
C17—C16—C21—C20	0.4 (3)	Sn2—C43—C48—C47	−178.21 (16)
Sn1—C16—C21—C20	177.70 (17)	C46—C47—C48—C43	−0.4 (3)
C10—Sn1—C22—C27	48.80 (17)	C37—Sn2—C49—C50	−153.78 (16)
C16—Sn1—C22—C27	−68.54 (17)	C43—Sn2—C49—C50	−42.98 (18)
S1—Sn1—C22—C27	−170.33 (14)	S3—Sn2—C49—C50	59.63 (18)
C10—Sn1—C22—C23	−136.57 (17)	C37—Sn2—C49—C54	18.37 (19)
C16—Sn1—C22—C23	106.10 (17)	C43—Sn2—C49—C54	129.18 (17)
S1—Sn1—C22—C23	4.30 (18)	S3—Sn2—C49—C54	−128.22 (16)
C27—C22—C23—C24	1.5 (3)	C54—C49—C50—C51	−0.5 (3)
Sn1—C22—C23—C24	−173.10 (17)	Sn2—C49—C50—C51	171.82 (19)
C22—C23—C24—C25	−0.3 (4)	C49—C50—C51—C52	−0.7 (4)
C23—C24—C25—C26	−1.3 (4)	C50—C51—C52—C53	1.1 (4)
C24—C25—C26—C27	1.8 (4)	C51—C52—C53—C54	−0.3 (4)
C25—C26—C27—C22	−0.7 (4)	C52—C53—C54—C49	−1.0 (4)
C23—C22—C27—C26	−1.0 (3)	C50—C49—C54—C53	1.3 (3)
Sn1—C22—C27—C26	173.95 (19)	Sn2—C49—C54—C53	−170.94 (19)

### Hydrogen-bond geometry (Å, °)

**Table:**

Cg1, Cg2, and Cg3 are the centroids of the C16–C21, C37–C42 and C43–C48 benzene rings, respectively.

**Table:**

*D*—H···*A*	*D*—H	H···*A*	*D*···*A*	*D*—H···*A*
C9—H9···Cg1^i^	0.95	2.72	3.630 (3)	160
C25—H25···Cg2^ii^	0.95	2.90	3.639 (3)	135
C32—H32···Cg3^iii^	0.95	2.92	3.824 (2)	160

Symmetry codes: (i) −*x*+1, −*y*+2, −*z*+1; (ii) *x*−1, *y*, *z*; (iii) −*x*+2, −*y*+2, −*z*+2.
